# Probiotics Administration in Cystic Fibrosis: What Is the Evidence?

**DOI:** 10.3390/nu14153160

**Published:** 2022-07-30

**Authors:** Susanna Esposito, Ilaria Testa, Elena Mariotti Zani, Daniela Cunico, Lisa Torelli, Roberto Grandinetti, Valentina Fainardi, Giovanna Pisi, Nicola Principi

**Affiliations:** 1 Pediatric Clinic, Department of Medicine and Surgery, University of Parma, 43126 Parma, Italy; elena.mariottizani@unipr.it (E.M.Z.); dani.cunico@gmail.com (D.C.); lisa.torelli92@gmail.com (L.T.); robertograndinetti93@gmail.com (R.G.); 2 Respiratory Unit, Great Ormond Street Hospital for Children, Foundation Trust, London WC1N 1LE, UK; ilariatesta@alice.it (I.T.); valentina.fainardi@unipr.it (V.F.); gpisi@ao.pr.it (G.P.); 3 Università degli Studi di Milano, 20122 Milan, Italy; nicola.principi@unimi.it

**Keywords:** cystic fibrosis, dysbiosis, gut-lung axis, microbiota, probiotic

## Abstract

In the last 20 years, gut microbiota in patients with cystic fibrosis (CF) has become an object of interest. It was shown that these patients had gut dysbiosis and this could explain not only the intestinal manifestations of the disease but also part of those involving the respiratory tract. The acquisition of previously unknown information about the importance of some bacteria, i.e., those partially or totally disappeared in the gut of CF patients, in the regulation of the activity and function of the gut and the lung was the base to suggest the use of probiotics in CF patients. The main aim of this paper is to discuss the biological basis for probiotic administration to CF patients and which results could be expected. Literature analysis showed that CF intestinal dysbiosis depends on the same genetic mutations that condition the clinical picture of the diseases and is aggravated by a series of therapeutic interventions, such as dietary modifications, the use of antibiotics, and the administration of antacids. All this translates into a significant worsening of the structure and function of organs, including the lung and intestine, already deeply penalized by the genetic alterations of CF. Probiotics can intervene on dysbiosis, reducing the negative effects derived from it. However, the available data cannot be considered sufficient to indicate that these bacteria are essential elements of CF therapy. Further studies that take into account the still unsolved aspects on how to use probiotics are absolutely necessary.

## 1. Introduction

Cystic fibrosis (CF) is a heritable, autosomal recessive disease caused by mutations in a gene located on the long (q) arm of chromosome 7 at position 31.2 that codes for the cystic fibrosis transmembrane conductance regulator (CFTR) protein, a small ion channel that is essential for sodium and chloride transport across cell membranes [1,2]. Currently, more than 2110 *CFTR* mutations have been identified [3]. Of them, only 401 are recognized as CF-causing mutations because they are associated with absent or decreased function of CFTR protein and development of CF [4,5]. This is a disease mainly characterized by thickened and poorly hydrated mucus secretions in various organs and body systems, including the respiratory tract, liver, pancreas, gallbladder, and intestine [6,7]. CF phenotypes can significantly vary according to the nucleotide alterations in the *CFTR* gene and the impact of environmental and additional genetic factors on CFTR-protein activity [7,8]. However, the most severe CF cases are those with the greatest respiratory tract involvement, as respiratory infections and lung functional deterioration are the most common causes of hospitalization and early death in CF patients [9]. For this reason, the majority of the microbiological studies carried out in CF patients since the identification of the disease have regarded respiratory microbiota and the impact of its modifications on the illness course, the risk of acute respiratory exacerbations, the choice of the most effective antibiotic therapy, the development of antibiotic resistance, and the potential therapeutic value of all the interventions capable of restoring the normal respiratory flora composition [10,11,12,13,14].

The CF gut microbiota was initially neglected. Only in the last 20 years has it become an object of interest, and only more recently it has been thoroughly studied [15,16]. Several factors may explain this. It was partially because of the evidence that gut microbiota composition could be associated with the clinical course of several intestinal and extraintestinal diseases and that reversing dysbiosis, i.e., the imbalance of gut microflora with a reduction in microbial diversity and emergence of potentially pathogenic bacteria, could be of benefit not only for some intestinal problems but also for treatment of obesity, allergic disorders, type 1 diabetes mellitus, autism, and some respiratory conditions [17]. Even more important is the finding that CF patients themselves had dysbiosis and that this could explain at least in part not only the intestinal disorders but also the respiratory ones. The acquisition of previously unknown information about the importance of some bacteria, i.e., those partially or totally disappeared in the gut of CF patients, in the regulation of the activity and function of both the gut and the lung was the base to suggest the use of these microorganisms, the so-called probiotics, in CF patients [18,19]. Main aim of this paper is to discuss which are the biological basis for probiotic administration to CF patients and which results could be expected.

## 2. Characteristics of Gut Microbiota in Cystic Fibrosis

Several studies carried out in both experimental animals and humans have shown that individuals with CF have significant differences in gut microbiota composition compared to healthy subjects. Despite differences between studies, in CF animals and patients, gut bacterial diversity is generally decreased [20,21,22]. Moreover, potentially pathogenic microbes, such as those usually associated with gut infections, are expanded, whereas potentially beneficial bacteria, such as *Lactobacilli* and *Bifidobacteria,* are reduced [20,23,24,25,26]. In CF individuals, dysbiosis is established in the first weeks of life. Contrarily to healthy subjects in whom gut microbiota develop rapidly reaching definitive composition between 3 and 5 years of age, in CF children gut microbiota maturation is significantly slower. Diversity increases very slowly, remaining lower than in healthy subjects throughout adolescence. Moreover, gut microbiota composition is differently established [27,28,29]. Kristensen et al. compared gut microbiota of healthy and CF infants examining stool samples collected during the first 18 months of age [27]. They found that most healthy children initially had a *Bifidobacterium* dominated profile which was changed between month 8 and month 18 to a profile dominated by both *Bifidobacterium* and *Blautia*. On the contrary, although most CF children also started with a prevalent presence of *Bifidobacterium*, a relevant part of them was already colonized by *Streptococcus* or *Escherichia coli* from one month of life [27]. Moreover, more detailed analysis of gut microbiota composition revealed that several beneficial bacteria were more abundant in healthy children than in CF patients. This is the case for *Clostridia* IV and *Anaerostipes* [27] that, as well as *Bifidobacteria,* are short-chain fatty acid (SCFA) producing microbes [28] and *Akkermansia* degrades mucus [29]. On the contrary, in fecal samples of CF infants, bacteria strongly associated with inflammation, such as *E. coli* and *Veillonella,* were more frequently detected [27].

Similar gut microbiota modifications were also found in adult subjects [30,31], although it is not definitively established whether differences between CF patients and healthy subjects tend to increase progressively with age. However, as reported by Burke et al., compared to healthy subjects, adult CF patients generally have a significant increase in Firmicutes and a reduction in Bacteroidetes [24]. Moreover, differences were strictly related to the severity of CF manifestations. Subjects with severe lung dysfunction (% predicted FEV1 ≤ 40%) had significantly (*p* < 0.05) reduced gut microbiota diversity compared to those presenting with mild or moderate dysfunction [24].

## 3. Main Factors Leading to Gut Dysbiosis in Cystic Fibrosis Patients

Gut dysbiosis commonly seen in CF patients is basically dependent on the reduced CFTR protein function. Several animal studies suggest this conclusion [32,33,34]. Compared to wild-type animals, CFTR knockout mice have significant small intestinal bacterial overgrowth [32]. Moreover, they show remarkable differences in gut microbiota composition due to a relevant decrease in bacterial community richness, evenness, and diversity [33]. Finally, it has been shown that when wild mice are colonized with CF-selected microbiota, they tend to revert to a non-CF microbiota, suggesting that the characteristics of gut microbiota of CF individuals are disease specific [34]. Data collected in humans further indicate that the presence of one or more *CFTR* mutations is associated with gut dysbiosis development, although a strict relationship between the type and number of mutations and severity of dysbiosis is not precisely defined [35,36]. Differences between studies in this regard can derive from the methodological limitations of some studies that did not consider the role of other genes and/or external confounding in conditioning results [35]. However, Schippa et al. reported that patients with severe phenotypic expression and homozygous delF508 mutations were those with the greatest gut microbiota modifications, with a reduction in beneficial bacteria, such as *Bifidobacterium* and *Faecalibacterium* species, and the greatest prevalence of pathogens, such as *E. coli* and *Eubacterium biforme* [36].

Several potential mechanisms have been suggested to explain why *CFTR* dysregulation modulates the gut microbiome. The chloride channel dysfunction leads to thick, and inspissated mucus that accumulates along the villus surfaces and is slowly cleared [37], so providing an anchorage for bacterial adherence and abnormal colonization. The malabsorption of dietary nutrients, mainly the fat component, is associated with the emergence of certain microorganisms that fit better with the different environmental characteristics. It has been shown that *E. coli* detected in feces of CF patients differ from those cultured in healthy subjects as they present peculiar metabolic characteristics that are considered an adaptation to the greater availability of intestinal fats [38]. The reduced bile acid production and the decreased secretion of pancreatic enzymes and bicarbonate lead to a lower pH of the intestinal content. This can directly select gut bacteria or indirectly favor dysbiosis through the reduction in intestinal antimicrobial peptides [31,39]. Paneth cells secrete a variety of MyD88-dependent antimicrobial compounds that protect the epithelium and trapping of the Paneth cell granules in the crypts could alter their antimicrobial activity in the CF gut [40].

Together with *CFTR*-related factors, several acquired factors can further modulate gut microbiota composition, leading to dysbiosis. Antibiotic administration is the most common and the most important. Antibiotics are largely prescribed to CF patients as they are essential for the prevention and treatment of the numerous respiratory tract infections that continuously undermine the health of these subjects [41,42,43,44]. Unfortunately, their use has been repeatedly found to be associated with the same modifications of gut microbiota composition that characterize CF, with reduced presence of *Bacteroides* and *Bifidobacterium,* and increased concentrations of *Enterococcus* [27,45,46,47]. Moreover, although dysbiosis can occur even after short-term antibiotic exposure [48,49,50], it is more common and severe when repeated courses of antibiotic therapy are prescribed. CF individuals receiving the greatest exposure to antibiotics because given repeated intravenous antibiotic courses had the lowest microbiota diversity [24].

Diet is a second factor that may lead to gut dysbiosis in CF patients, although the relationships between diet composition and dysbiosis development are not definitively clarified. Fat content seems the most relevant factor. Due to fat malabsorption and risk of poor nutrition, CF patients are given high-fat diets [5,7]. In mice, this type of diet has been negatively associated with the abundance of *Akkermansia* but positively associated with the relative abundances of *Firmicutes* and *Allobaculum*. Starting from animal studies, it has been hypothesized that high-fat diets can favor translocation of some bacterial communities or increase abundance of lipopolysaccharide-producing bacteria also in humans [51].

Finally, use of proton pomp inhibitor (PPI), i.e., drugs frequently administered to CF patients to face gastroesophageal reflux disease may play a role in favoring gut dysbiosis development in CF patients. Although there are some exceptions [31], studies regarding use of these drugs in the general population have shown that it can be associated with significant gut dysbiosis, favoring the increase in pathogenic bacteria. Lower species diversity and overrepresentation of *E. coli*, *Enterococcus* spp., and *Streptococcus* can be commonly found in stool samples of these patients [52]. Moreover, association between increased risk of gut infections due to *Clostridium difficile*, *Salmonella* spp., *Shigella* spp., and *Campylobacter* spp., and PPI use has been repeatedly reported [53,54].

## 4. Impact of Gut Dysbiosis in Cystic Fibrosis Patients

Gut dysbiosis of CF patients significantly contributes to the severe intestinal inflammation and altered intestinal structure and function seen in many CF patients. Most bacteria that are significantly reduced in CF gut microbiota, such as those included in the *Ruminococcaceae* family (mainly *F. prausnitzii* [55]), in the *Bifidobacteria* genus [56], and in the *Lactobacillus* genus [57] exert, among others, also have relevant anti-inflammatory properties. On the contrary, some of the pathogens, whose concentration is significantly increased in CF gut microbiota, such as *E. coli*, *Clostridium perfringens*, *S. aureus*, and *P. aeruginosa*, are directly associated with gastrointestinal inflammation [58]. Evidence of gut inflammation in CF patients has been reported since 2000, when Smyth et al. showed that CF patients had increased intestinal outputs of albumin, IgG, IgM, eosinophil cationic protein, neutrophil elastase, interleukin 1beta (IL-1β), and interleukin 8 (IL-8) [59]. Moreover, in the same year, Raia et al. reported that, in children with CF, mononuclear cells in the lamina propria of the intestinal mucosa had an increased expression of several immunological markers, suggesting the activation of an immune mediated inflammatory process [60]. Later studies have confirmed these findings, highlighting that CF patients suffer from a condition similar, although less severe, to that usually found in patients with inflammatory bowel diseases. Elevated fecal concentrations of calprotectin, M2-pyruvate kinase, and rectal nitric oxide, all markers of gut inflammation, are commonly found in CF patients [61,62,63,64,65,66].

Chronic inflammation significantly impacts on CF patient growth. Fecal calprotectin concentrations have been found strictly associated with the degree of underweight and the reduction in weight and height z-scores [67]. Moreover, gut inflammation seems to influence CF severity, including risk of lung infection and progressive deterioration. In a systematic review of studies regarding fecal calprotectin and phenotype severity in CF patients, Talebi et al. reported that there were significant correlations between fecal calprotectin and the factors that characterize a more severe CF phenotype, including colonization by *P. aeruginosa*, predicted FEV1 <50%, pancreatic insufficiency, and underweight status [68]. Moreover, reduced gut microbiota diversity has been found associated with lower FEV1 values [23] and increased risk of pulmonary exacerbations [25]. All these findings seem to indicate that the gut microbiota is related to lung function through the so-called gut–lung axis that allows homeostasis in both compartments. Practically, it is thought that, mainly through gut microbiota-derived metabolites, gut beneficial bacteria can regulate immune response at both the intestinal and respiratory level (see later the section on probiotics for detailed mechanisms). Several data support this relationship. In experimental animals, gut microbiota influences the risk of respiratory tract infection development [69]. Early gut dysbiosis is associated with increased risk of atopy and asthma development [70,71,72].

Patients with chronic gastrointestinal diseases, such as irritable bowel syndrome (IBS) and inflammatory bowel disease (IBD), have a higher prevalence of pulmonary diseases [73,74,75]. Regarding CF patients, it was evidenced that microbial communities of the gut and lung develop simultaneously and share several colonizing species [76]. Moreover, the appearance of some species in the gut is associated with the following appearance of the same species in the lungs, suggesting that the gut microbiota may help shape the development of the lung microbiota [70,77].

Furthermore, it has been reported that colonization of the respiratory tract with *P. aeruginosa*, a marker of severe respiratory disease, are preceded by a significant reduction in gut beneficial microbes, mainly *Parabacteroides* [1].

Finally, gut dysbiosis is considered a significant contributor to the development of gut malignancies that are significantly more common in CF patients that in individuals without CFTR mutations [78,79].

## 5. Mechanisms of Probiotic Potential Benefits

According to the Food and Agriculture Organization (FAO)/World Health Organization (WHO) working groups, probiotics are defined as live microorganisms that, when administered in adequate amounts, confer a health benefit to the host [80]. Initially, probiotic preparations were mainly based on lactic acid producing bacteria, such as *Lactobacilli* and *Bifidobacteria*. More recently, other microorganisms capable of inducing beneficial effects were identified. Among them, bacteria included in *Eubacterium, Propionibacterium, Faecalibacterium, Akkermansia*, and *Roseburia* species are the most frequently used [81,82].

How probiotic administration can lead to gastrointestinal and extraintestinal benefits is not precisely defined, although several contributing factors have been suggested. It has been shown that probiotics can significantly influence intestinal colonization by bacterial pathogens, favor maintenance of structural integrity of the gut mucosal barrier, regulate production of beneficial metabolites, modulate immune system activity, and stimulate vitamin production [70]. Unfortunately, effects can significantly vary from probiotic to probiotic and can be different according to various factors, such as the dose administered, the duration of administration, and the substrate on which they act. These findings explain, at least in part, why evaluation of clinical efficacy of probiotic administration in the different conditions in which they could be prescribed remains very difficult. Competition for nutritional sources and production of bacteriocin or bacteriocin-like substances are the most important mechanisms with which probiotics can inhibit intestinal pathogenic bacteria and displace them from the gut [83]. In vitro studies have shown that *Bifidobacterium lactis* Bb12 and *Lactobacillus rhamnosus* LGG, alone and in combination, could inhibit the adhesion of pathogenic strains, such as *Salmonella, Clostridium*, and *E. coli,* to pig intestinal mucosa [84]. Similarly, it was found that *L. paracasei* FJ861111.1 could strongly inhibit multiplication and adhesion to intestinal cells of common pathogens, including *Shigella dysenteriae, S. aureus, Cronobacter sakazakii, E. coli*, and *Candida albicans* [85]. Most of these findings were confirmed in the experimental animal [86] and are one of the most important reasons for the suggestion to use probiotics for the prevention and treatment of some human diarrheal diseases [87].

Improvement of gut barrier integrity and function derives from the ability of probiotics of regulate tight junction functions of epithelial cells and mucus properties. *Lactobacillus* and *Bifidobacterium* strains have been found able to increase the expression of tight junction proteins so preventing translocation of proinflammatory substances and pathogenic bacteria [88,89,90]. Moreover, the same bacteria can upregulate mucin production genes, reducing the risk the pathogenic bacteria can adhere to the intestinal cells [91,92,93].

Influence of probiotics on host metabolism is evidenced by the role of these bacteria on bile acid metabolism, short chain fatty acid (SCFA) production, and reduction in plasma lipopolysaccharide concentration. Gut microbiota deconjugate bile acids (BAs) through the action of microbial enzymes. These are produced by various microbial species, including those in the *Lactobacillus, Bifidobacterium, Enterococcus*, and *Clostridium* genera [94]. Deconjugation results in increased gut concentrations of antimicrobial BAs, cholic acid, and chenodeoxycholic acid that have significant antimicrobial activity and may drive shifts in microbiome composition [95]. Some probiotics, such as *Lactobacillus plantarum*, have a greater resistant than some pathogenic bacteria to BA-induced bacterial membrane lysis and can have a significant role in favoring pathogen elimination and dysbiosis reduction [96].

SCFAs derive from the microbial fermentation of dietary fibers and include acetate, propionate, and butyrate. Acetate production is common to several bacterial groups and seems important because of its role in the lipid and glucose metabolism favoring protection from fat accumulation and improving glucose tolerance [97,98]. Similar, but greater, metabolic effects have been reported for propionate that, in addition, seems to induce a protective effect in the gastrointestinal tract, reducing the risk of cancer development. It is produced by relatively few bacterial genera, among which *Akkermansia municiphila* is the most important [99]. The most relevant SCFA is, however, butyrate that is mainly produced by *F. prausnitzii, Eubacterium rectale, Eubacterium hallii* and *Ruminococcus bromii* [100]. This SCFA, together with very relevant positive influence on lipid and glucose metabolism, exerts strong anti-infective and anti-inflammatory properties at the gut level [101].

Probiotics can reduce plasma lipopolysaccharide (LPS) concentration and the risk of severe metabolic derangements, including metabolic endotoxaemia, that can follow LPS accumulation. LPS is the major glycolipid component of the outer membrane of Gram-negative bacteria, that are significantly increased when gut dysbiosis occurs and that can be competitively reduced by probiotics. LPS accumulation is associated with a relevant increase in intestinal permeability, severe gut inflammation, and the risk of metabolic endotoxaemia with the emergence of glucose intolerance, hepatic insulin resistance, and fat accumulation [102].

Probiotics can directly modulate immune system activity, and this can have effect not only at the intestinal level but also in several extraintestinal sites, including the lungs. When pattern recognition receptors on host epithelial and immune cells recognize microorganism-associated molecular patterns expressed by probiotics, a molecular response against these bacteria is generated. Cytokines and chemokines are produced and can enter the blood circulation reaching distant organs, including the lungs, where they can exert their specific activity [103]. Moreover, probiotics are internalized by gut dendritic cells and, through these, promote the activation of various T cell subsets and the production of various cytokines. T cell subsets then acquire immune homing molecules that allow their function in extraintestinal organ and body systems [104].

Finally, it has been suggested that a direct microbial translocation from gut to lung may occur. As colonization of gut in infants is associated with similar colonization in the lung, it has been supposed that similar lung modifications with beneficial effects could occur when probiotics revert gut dysbiosis [39,105].

## 6. Probiotic Use in Cystic Fibrosis Patients

Several studies were carried out with the intent to evaluate whether oral probiotics could reduce gut inflammation and respiratory symptoms of CF patients. Unfortunately, results of presently available clinical trials are conflicting and do not allow to definitively establish whether probiotics are effective in reducing CF manifestations [106,107,108,109,110,111]. In most of the studies, significant effects on the frequency of respiratory infections and intestinal inflammation markers, mainly calprotectin levels, were reported. However, discrepant results of poor or no effect also exist. Moreover, in some studies emergence of adverse events, including vomiting, diarrhea, and allergic reactions, were reported. All these findings explain why no guideline, including those produced by the European Society for Clinical Nutrition and Metabolism (ESPEN), the European Society for Pediatric Gastroenterology Hepatology and Nutrition (ESPGHAN), and the European Cystic Fibrosis Society (ECFS) [7], considers probiotics among treatment measures for CF patients, and in many countries probiotics are not licensed as drugs but are simply considered as food supplements.

Detailed descriptions of probiotic impact on people with CF have been reported in the most recent systematic Cochrane review that includes 12 completed randomized controlled trials and 1 trial that was terminated early [111]. Both children and adults were enrolled for a total of 464 participants. Data were analyzed combining studies with the same primary objectives. When the number of pulmonary exacerbations during a 4–12 month timeframe was considered [2,112,113,114], it was calculated that patients receiving a probiotic had suffered from 0.32 (95% confidence interval (CI) −0.68 to 0.03) fewer episodes than controls given placebo. This difference was not statistically significant (*p* = 0.07) and the 95% CI suggests that probiotic administration could be associated with both a positive or a negative effect on the frequency of respiratory exacerbations. No evidence of a positive effect was shown when lung function, hospitalization rates, and use of antibiotics were studied. Finally, when the effects on gut inflammation and growth were studied, low-certainty evidence of a positive effect or no effect were reported. Several factors may explain the discordant results. Intrinsic methodological limitations that make results debatable can be detected in some trials. Moreover, studies are not comparable. The number of people enrolled in each study varied from 22 to 81, both children and adults with different disease severity were included, probiotic preparations varied in type, dosage, and length of administration. In this regard it must be highlighted that, while *Lactobacilli* were mainly used, the dose varied from 10^8^ colony forming unit (CFU)/day to 10^11^ CFU/day and duration ranged from 1 month to 1 year. In some cases, multistrain formulations with prebiotics were used [2,112,113,114,115,116,117].

The limitations mentioned above clearly indicate that further studies are needed before probiotics administration can be considered an indispensable therapeutic intervention for CF treatment. New prospective, larger, and well-designed studies are needed. According to Neri et al. [107], study protocols should precisely define age and characteristics of the enrolled population as age can be an important interference factor. Moreover, the positive effect of treatment should be evaluated, taking into account that previous studies have shown that frequency of pulmonary exacerbations and reduction in intestinal inflammation markers were the most reliable targets to establish probiotic efficacy but that a minimum reduction in these variables sufficient to define improvement should be a priori established. Finally, long-term therapy should be planned. However, it seems likely that, even when studies considering these suggestions will be performed, not all the problems related to the use of probiotics in CF patients will be solved. Timing of the intervention according to clinical condition of CF patients will remain undefined and totally undefined is the role of the CFTR modulators, recently entered in the CF therapy, on gut dysbiosis modulation. Theoretically, subjects with CFTR mutations strongly associated with the most severe disease phenotype should receive probiotics as soon as genetic testing is available, which presently does not always happen. The choice of the best strain or combination of strains remains a problem, particularly when probiotics are given not only to have a generic effect on gut and lung microbiota but to target specific CF pathogens. Probiotic effects are largely species and strain specific and some probiotics, despite being included in the same genus, can have different antimicrobial activity against CF pathogens. For example, *Lactobacillus* strains, alone or in combination, are frequently the base for several probiotic preparations, but the choice of one or the other could depend on the fact that *Pseudomonas aeruginosa* is the target of the intervention because the antibacterial activity against this pathogen can significantly vary among strains [118]. Ability of gut and lung colonization of different probiotics remains unknown. Presently, marketed probiotics are selected taking into account their ability to reach the intestine and adhere to epithelial cells, but it is not defined if bacteria transferred from gut to lung can long survive in this environment and play a role in exerting beneficial activity [50].

## 7. Conclusions

In CF, intestinal dysbiosis is extremely common. It depends on the same genetic mutations that condition the clinical picture of the disease and is aggravated by a series of therapeutic interventions, such as dietary modifications, the use of antibiotics, and the administration of antacids. All this has profound practical repercussions because, alongside chronic inflammation, the imbalance of the composition of the enteric flora is associated with an alteration of the integrity of the intestinal barrier, with negative modifications of the immune response and with an altered functionality of the intestine–lung axis. All this translates into a significant worsening of the structure and function of all organs, including the lung and intestine, that are already deeply penalized by the genetic alterations of CF. There are experimental data and some studies in humans that probiotics can intervene on dysbiosis, reducing the negative effects derived from it. The available data cannot, however, be considered sufficient to indicate that these bacteria are essential elements of CF therapy. Further studies that take into account the still unsolved aspects of how to use probiotics are absolutely necessary. However, to be really effective, these studies should be planned with particularly attention, taking into account what has been already demonstrated and which problems should be necessarily solved. Finally, the role of the CFTR modulator activity should be considered.

## Data Availability

All the data are included in the manuscript.
